# Sex-Specific Immune Responses to Seasonal Influenza Vaccination in Diabetic Individuals: Implications for Vaccine Efficacy

**DOI:** 10.1155/2023/3111351

**Published:** 2023-10-17

**Authors:** Anirban Sengupta, Noha Al-Otaibi, Jorma Hinkula

**Affiliations:** ^1^Department of Biomedical and Clinical Sciences, Linköping University, Linköping 58185, Sweden; ^2^King Abdulaziz City for Science and Technology (KACST), Riyad 11442, Saudi Arabia

## Abstract

Seasonal influenza vaccination has different implications on the immune response depending on the comorbidities. Diabetes is one such critical disease that increases the patient's susceptibility to influenza and suppresses vaccine efficacy and immunity. The sex of the individuals also plays a definitive role in the immune responses to both the vaccine and the infection. This study aims to understand the efficacy of the seasonal vaccine against influenza in diabetic groups and undergoing immune mechanisms in different sexes (females and males). In this study, we are reporting about a switching of the immune response of the infected and vaccinated diabetic females towards stronger Th1/Th17 responses with suppressed humoral immunity. They show increased cDC1, enhanced proinflammatory activities within T cells, CD8T activation, Th17 proliferation, and the majority of IgG2 antibody subtypes with reduced neutralization potential. Males with diabetes exhibit enhanced humoral Th2-immunity than the nondiabetic group. They exhibit higher cDC2, and DEC205 levels within them with an increase in plasma B lymphocytes, higher IgG1 subtypes in plasma cells, and influenza-hemagglutinin-specific IgG titer with stronger virus neutralization potential. Males with diabetes recovered better than the females as observed from the changes in their body weight. This study highlights the critical immune mechanisms and sex-specific swapping of their preferred immune response pathways against influenza after vaccination during diabetes. We propose a need for a sex-specific customized vaccine regimen to be implemented against influenza for individuals having diabetes to exploit the manifested strength and weakness in their protective immunity.

## 1. Introduction

Influenza is one of the notorious viruses affecting humankind for centuries. The virus and its impact on our immune response have been extensively studied for decades. Novel vaccination strategies are being developed worldwide to prevent or curb pandemics of this respiratory virus. However, one major factor generally overlooked in this vaccine development process is the sex of the recipients. It has been extensively studied and reviewed [1, 2] that male and female shows quite a difference in their immunity and their response to the vaccination [3, 4]. Even though a growing body of literature highlights the sex-based differences in immunity, immunology ranked in the lowest position out of the 10 major biological disciplines for mentioning the sex of the participant human or animal model [5, 6]. Over 90% of the immunology papers do not analyze their data by sex [5, 6].

In 2010, the WHO published a report providing detailed evidence of how sex and gender play a critical role in the outcome of influenza virus infection [7]. They highlighted the poor outcome of the adult female patients with the infection. More deaths in adult females have been reported in the United States during the 1957 H2N2 pandemic [8], worldwide during H5N1 infection in 2008, worldwide in 2009 H1N1 pandemic [9] specially reported in Canada [10], Japan [11], and China [12]. Several reports and lab studies are suggesting the greater pathogenesis and poor host outcome of influenza infection in females [13, 14]. In a murine model, the studies on sex differences in the immune response against influenza infers the females are more susceptible and more likely to succumb to infection in lower viral doses than the males [15, 16].

The varied impact of influenza viral infection on the host also depends on the comorbidities the individual may have pre- or post-infection. Diabetes is one such predominating disease that has a critical impact on host immunity against influenza [17, 18]. Studies have shown the increased susceptibility of the diabetic patients to severe infection due to impaired immune response [19, 20]. Many recent studies reported diabetes-associated immune pathology with increased disease severity, and mortality in influenza infection [21, 22]. Studies have attempted to address the issue but still the puzzle remains unanswered [18].

One common mode of protection against influenza is the seasonal vaccination program. However, its efficacy is debatable in the diabetes group [18]. Two small retrospective studies reported that the pandemic influenza in 2009 is associated with Type 1 diabetes [23, 24]. These studies reported increased diabetic pathogenesis or severity for those infected with H1N1 virus during the pandemic [23, 24]. Diabetes is reported to suppress and alter the immune response and also modify the vaccine response [17, 18, 25]. Higher blood glucose levels have a broad impact on immune metabolism and function [26]. Some of the studies reported the efficacy and benefits derived from the seasonal influenza vaccination in diabetic patients [18, 27–29]. These studies are mostly based on observational studies where clinical endpoints like hospitalization and death, beneficial reduction of morbidity and mortality, or correlation with diabetes and influenza severity and death were reported. Other studies only focus on the serological antibody titer and its correlation with the outcome and did not cover the cellular response. There is a huge knowledge gap about the mechanism played by immune cells in shaping such response or outcome.

We evaluate the immune response from an all-around study comprising an infection (influenza) and its vaccination (VaxiGrip) response along with comorbidity (diabetes) in different sexes (male and female). In this study, we answered the long-standing question of how diabetes plays a crucial role in influencing our immune system during influenza vaccination, with a focus on the sex differences and their impact on the humoral and adaptive immune response derived from the dendritic cells, T and B lymphocytes, and the antibody response.

## 2. Materials and Methods

### 2.1. Animal Use and Experiment Groups

Male and female mice, 6–8 weeks old C57Bl/6 were obtained from Janevier, Sollentuna, Sweden. The mice were divided into six experimental groups (*n* = 5 for each group), including two control groups: male control (MCtrl) and female control (FCtrl). Human seasonal influenza vaccine Vaxgrip was provided subcutaneously to four groups ((the diabetes groups; FD+, MD+), (normal/nondiabetic groups; FD− and MD−)) along with human endogenous lipid emulsion adjuvant derivative of the N3 (cationic) lipid formulations obtained from Merck (Darmstadt, Germany). This adjuvant has been used in our previous studies as well [30–32] and is well-established for boosting immunity. Influenza viral challenge was provided in all four groups of vaccinated mice. The viral challenge has also been administered to another group of six mice (three males and three females) which are unvaccinated. All of these six mice were dead within 2 weeks of the challenge and were excluded from the study. The control groups of the study (MCtrl and FCtrl) received the same amount of saline solution instead of the virus and they survived till the end of the experimental timeline. All the mice from these two control groups and the other four groups (FD−, FD+, MD−, and MD+) were sacrificed on the 31st-day postviral challenge date. The animals received a standard rodent diet. Food and water were available ad libitum. The water supply was changed daily. The cages were changed weekly. The change in the physical parameters is checked and recorded. This study was carried out in strict accordance with the recommendations in the Guide for the Care of Laboratory Animals at the Linköping University. The protocol was approved by the Committee on Ethics of Animal Experiments of the Linköping University (Protocol Number Dnr 18053-2020 and Dnr: 00234-2022).

### 2.2. Inducing Diabetes in the Mice Group

The mice in group FD+ and MD+, were injected intraperitoneally with streptozotocin (Sigma–Aldrich) after dissolving it in 50 mM sodium citrate buffer (pH 4.5). The dose and the procedure were the same as done before [33]. A amount of 60 mg/kg dose was provided for consecutive 3 days while other groups got an equal amount of vehicle. Two weeks after the treatment fasting blood glucose was measured with a glucose analyzer (Acc-check). All the mice considered in the study have their blood glucose level above 17 mmol/L as done before [33].

### 2.3. Immunization and Infection Challenge

**Figure 1:**
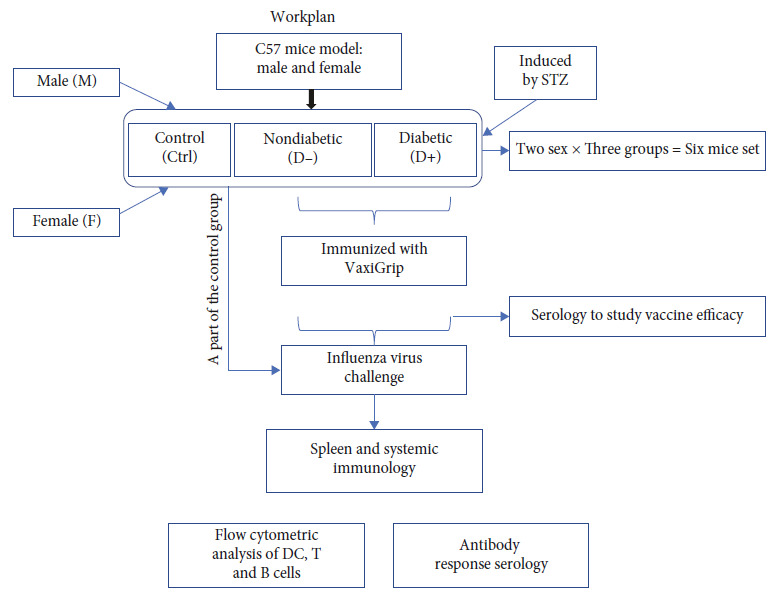
The schematic representation of the workflow.

Mice received 1 *μ*g of Vaxigrip HA/mouse with cationic adjuvant N3 (Eurocine). During vaccination, the mice were anesthetized with isoflurane (IsoFlo® vet, Orion Pharma Animal Health, Sollentuna, Sweden). The mice received three shots at different intervals (days 0, 21, and 42). Blood samples were collected in between respective doses from the vena saphena to measure the generated immune response in the animals. After 28 days of the last vaccination, the mice were challenged with the influenza virus (influenza A/H1N1/California/07/2009), and 10 *μ*l of challenge dose 50LD_50_ was administered intranasally. A separate seventh group of six unvaccinated mice (three males and three females) were also challenged with the virus. All of the six mice in this group died within 2 weeks of the viral challenge and were thus excluded from the study (and hence not mentioned in Table 1). All the remaining six mice groups (as mentioned in Table 1) were sacrificed on the 31st-day postviral challenge or postmock challenge (for the MCtrl and FCtrl). No death of the mice were observed in all of these six groups mentioned in Table 1. So the subsequent study and the analysis were done on *n* = 5 for each of the six groups. The detailed work plan discussed here is provided in the schematic representation format in the flowchart provided in Figure 1. The body weight of mouse in the experiment was measured daily to monitor the changes.

### 2.4. Flow Cytometry

Flow cytometry on the splenic single-cell suspension was done as described before [34] and also discussed in detail in the supplementary methods section. The use of the antibodies combination for determining the cellular subsets, phenotypes, activation, and so forth along with the references are also provided in Table S1. Briefly, the splenic single-cell suspension from the sacrificed mice was subjected to washing and blocking followed by fluorochrome-tagged antibody incubation. For intracellular staining cellular permeabilization was done followed by fixing. Isotype-matched antibody controls were taken. The data compensation of the fluorescence spillover was performed. Fluorescence signals from the labeled cells were acquired using a BD AriaIII Machine and analyzed by FlowJo software.

### 2.5. Serology Screening

ELISA was performed on the serum separated from the blood samples (0.5 ml) that were collected from all the animal groups in Table 1 on the day of sacrifice following the standard procedure described before [31] and in the supplementary methods section. Briefly, recombinant H1N1 or H3N2 hemagglutinin (HA) antigen was used to coat the ELISA plates followed by serum sample incubation, washing, and HRP-labeled conjugate goat-antimouse IgG (BioRad, Richmond, CA) antibody incubation. For the IgG subtyping, antimouse IgG1, IgG2a, IgG2b, and IgG3 antibodies are used followed by antigoat IgG-HRP antibody. *o*-Phenylenediamine (from Sigma–Aldrich, St. Louis, MA) solution is used to develop the signal followed by reaction termination by H_2_SO_4_. The absorbance was measured at OD in an ELISA plate reader (Molecular Devices, Spectramax ID3). The cutoff value for positive reactivity was calculated from the mean OD_490_ plus SD for negative control samples.

### 2.6. Hemagglutination Inhibition Assay

The hemagglutination inhibition assay (HAI) was used to evaluate the presence of neutralizing anti-HA antibodies against viral influenza A in serum from individual mice as described previously [35] and in the supplementary methods section. The HAI assay was initiated by adding 25 *µ*l phosphate-buffered saline to each well of a microtiter plate, followed by the addition of 50 *µ*l of the receptor-destroying enzyme (RDE) treated serum. The serum was diluted in eightfold serial dilutions starting from 1/10 up to 1/1,280 dilutions. A amount of 25 *µ*l of influenza A/H1N1/CA09pdm containing four hemagglutinating units was added into each well. Subsequently, 50 *µ*l chicken erythrocytes were added, and mixed, followed by incubation at 4°C for 1 hr. Thereafter, the plate was evaluated for hemagglutination and the degree of HAI.

### 2.7. Statistical Analyses

Data analysis was performed using GraphPad Prism. Initial analysis of immunological parameters was performed using Kruskal–Wallis one-way ANOVA to understand the significant difference between the groups. The second independent comparisons between study groups were performed with nonparametric methods using the Mann–Whitney *U* test. *p* < 0.05 was considered statistically significant. *p* < 0.05 were considered significant ( ^*∗*^*p* < 0.05,  ^*∗∗*^*p* < 0.01, and  ^*∗∗∗*^*p* < 0.001). Only within-test corrections were made and not wide/across-test corrections. The pairwise comparison between individual groups was performed with Mann–Whitney *U* test using nonparametric method. The *p*-value asterisk shown in each figure of this manuscript is based on this Mann–Whitney test result.

## 3. Results

### 3.1. Higher cDC1 with Increased Costimulatory CD80/86 and MHCI Expression in the Female Diabetic Group as Compared to the Sex-Matched Nondiabetic Group Observed, Male Diabetic Group Showed an Increased cDC2 Population

**Figure 2:**
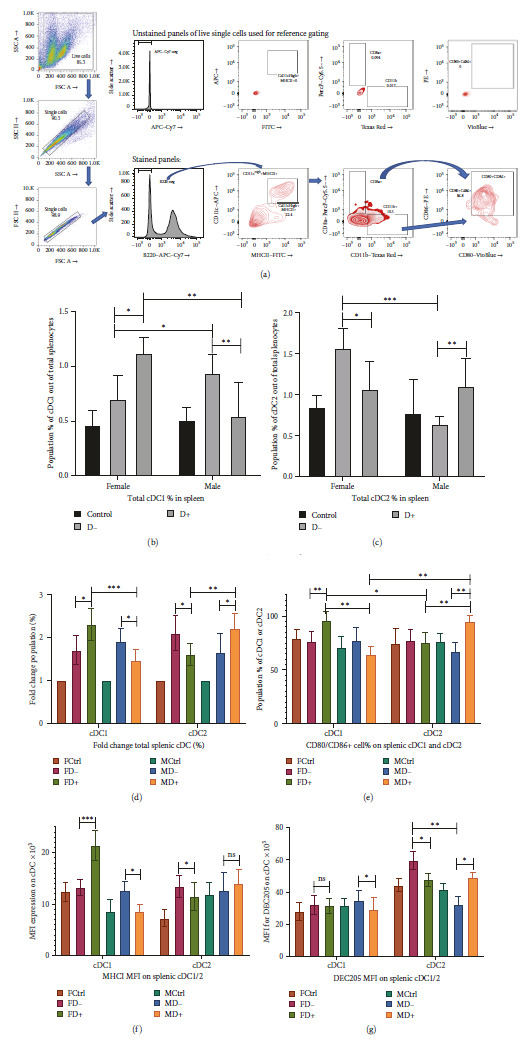
The evaluation of subsets of conventional dendritic cells (cDC). (a) Live single splenic cell suspension was gated for B220/CD45R negative population on which the MHCII+CD11c+ subset had been gated out. On that, CD8a+CD11b− and CD8a−CD11b+ cDCs were gated out to get B220−MHCII+CD11c+CD11b−CD8a+ (cDC1) and B220−MHCII+CD11c+CD11b+CD8a− (cDC2) population percentage. CD80+CD86+ positive cDC1 and cDC2 population percentages have also been analyzed. (b) The population percentage of cDC1 and (c) cDC2. The percentage exhibited in the *Y* axis is the total percentage within the 10,000 splenocytes analyzed. Hence, for absolute number conversion of this cDC percentage: 1% on *Y* axis = 100 cDC. (d) The fold change of population percentage of cDC1 and cDC2. (e) The level of CD80/86 double-positive cDC1 and cDC2 population percentages have been determined. (f) Mean fluorescence intensity (MFI) of MHCI and (g) DEC205 on cDC1 and cDC2 populations analyzed for determining the expression level. D−, nondiabetic groups; D+, diabetic groups. Data in graphs are the representative images derived from at least four independent experiments ( ^*∗*^*p* < 0.05,  ^*∗∗*^*p* < 0.01, and  ^*∗∗∗*^*p* < 0.001). For each group *n* = 5, the error bar indicates the standard deviation.

The evaluation of the population percentage and activation of myeloid lineage dendritic cells: B220−MHCII+CD11c+CD11b−CD8a+ conventional dendritic cell 1 (cDC1) and B220−MHCII+CD11c+CD11b+CD8a− conventional dendritic cell 2 (cDC2) [36, 37] revealed an interesting variation between males and females (Figure 2(a)–2(c)). A significant increase of cDC1 in the diabetic female group as compared to its nondiabetic counterpart (Figure 2(b)) with the exact opposite trend for cDC2 population percentage is observed (Figure 2(c)). The males, on the other hand, showed a decrease in cDC1 postdiabetes (Figure 2(b)). cDC2 level however increased significantly in the male diabetic group as compared to nondiabetic MD− (Figure 2(c)). The fold change in cDC1 and cDC2 population percentage also shows the same trend (Figure 2(d)) with an increase in cDC1 in diabetic females and an increase in cDC2 population in diabetic males, reversing from low cDC1 in nondiabetic females and high cDC2 in nondiabetic males.

The analysis of the cDC for the costimulatory marker expression CD80 and CD86 (Figures 2(a) and 2(e)) shows a significant increase of CD80+CD86+ cDC1 population in female diabetics (FD+) compared to FD− and MD− and MD+ (Figure 2(e)). A significant increase of CD80+CD86+ cDC2 is found in diabetic males as compared to MD−, FD−, and FD+ (Figure 2(e)). The absolute number of cell comparisons also reveals the same trend (Figures S1(a) and S1(b)). MHCI expression as observed by the mean fluorescence intensity (MFI) (Figure S2(a)) is significantly higher in FD+ cDC1 than in any other groups (Figure 2(f)). It is noteworthy that its level significantly dropped in MD+ as compared to MD− in cDC1 with no significant changes in cDC2 (Figure 2(f)). DEC205, expression is remarkably higher in FD− cDC2 as compared to all other groups (Figures 2(g) and S2(b)). Its level of cDC2 significantly dropped in diabetic females and significantly increased in diabetic males as compared to their sex-matched nondiabetic counterparts (Figure 2(g)).

A significant increase in cDC1 population in the female diabetic group with higher CD80+CD86+ activation markers and MHC1 expression indicates a potential for better CD8T cell activation. On the other hand, a significantly higher cDC2 population percentage in the male diabetic group with increased costimulatory marker CD80/86 and DEC205 expression indicates a better activation of CD4T-based humoral pathway.

### 3.2. CD8T and CD4T Cell Population Increased in the Female Diabetic Group as Compared to the Sex-Matched Nondiabetic Group

**Figure 3:**
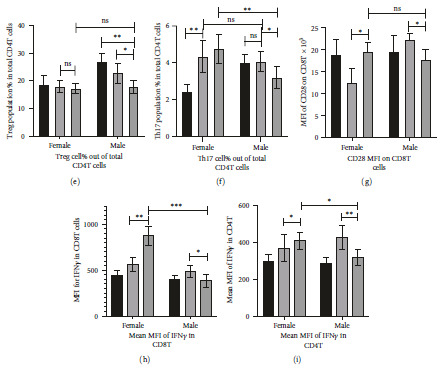
Evaluating the proportional and activity alterations in CD8T and CD4T cells and their subsets Treg and Th17 cell population. (a) The CD3+ cells are gated out from the live single splenocytes and the CD4T and CD8T populations are analyzed taking the unstained live single cells as reference gating. (b) The population percentage of cytotoxic CD8T cells and (c) the population percentage of helper CD4T cells were determined. The percentage exhibited in the *Y* axis is the total percentage within the 10,000 splenocytes analyzed. Hence, for absolute number conversion of CD8T or CD4T percentage: 1% in the *Y* axis = 100 cells. (d) The CD3+CD4+ T cell population gated out on the splenocytes (as in (a)) is analyzed for the expression of regulatory T cell (Treg) markers FOXP3 and Th17 cell marker RORgT. (e) Treg population (CD3+CD4+ FOXP3+) and (f) Th17 cell population (CD3+CD4+RORgT+) percentage of total helper CD4T cells were determined. The comparison between the absolute number of Treg and Th17 cells is provided in Figures S3(a) and S3(b). (g) The bar diagram of costimulatory marker CD28 expression on CD8T cells is determined by analyzing the MFI. The MFI and histogram plots are provided in Figure S3(c). (h and i) The MFI values were analyzed to determine the level of intracellular expression of proinflammatory cytokine of IFN*γ* as observed by flow cytometric staining of the permeabilized CD8T (h) and CD4T (i) cells, respectively. D−, nondiabetic groups; D+, diabetic groups. Data in graphs are the representative images derived from at least four independent experiments ( ^*∗*^*p* < 0.05,  ^*∗∗*^*p* < 0.01, and  ^*∗∗∗*^*p* < 0.001). For each group *n* = 5, the error bar indicates the standard deviation.

T cell subsets were studied by CD3 positive gating on live single-cell splenocytes followed by determination of CD4+ and CD8+ T cell population (Figure 3(a)). CD3+CD8+ cytotoxic T cell levels increased in FD+ as compared to FD−, while it decreases in MD+ as compared to MD− (Figure 3(b)). CD3+CD4+ Helper T cell population increased in FD+ with respect to FD−, with no significant change within the male groups (Figure 3(c)). Diabetes in males (MD+ group) showed a reduced CD4 helper T cell population as compared to diabetic females (FD+ group) (Figure 3(c)).

Overall, the increased CD8T population in diabetic females indicates a possibility of enhancement of CD8T-based cell-mediated immunity (Figure 3(b)). There is an overall reduction in both the CD4 helper T and CD8 cytotoxic T cell populations in all the infected groups as compared to their respective controls (Figures 3(b) and 3(c)). As our samples are collected on day 31 postinfection, there is a possibility of T cell apoptosis, exhaustion, and senescence as reported before in long-term infections [38].

### 3.3. Higher CD8T Activation Marker CD28 Expression, with an Increase of Th17 Cells Observed in Diabetic Female, Diabetic Male Exhibits a Significant Reduction of Both Treg and Th17 Cells

The CD3+CD4+ T cell population gated out on the splenocytes (as in Figure 3(a)) and analyzed for the expression of regulatory T cell (Treg) markers FOXP3 and Th17 cell marker RORgT (Figure 3(d)). While there is no significant change in Treg in female groups observed, their population dropped in the male diabetic (MD+) group (Figure 3(e)). There is also a significant increase in the Th17 cells in the female infected groups, especially during diabetes (Figure 3(f)). The absolute number of cell comparisons of both Treg (Figure S3(a)) and Th17 (Figure S3(b)) provides the same result as observed in the percentage comparison (Figures 3(e) and 3(f)).

While studying the costimulatory marker CD28 expression on CD3+CD8+T cells, showed significantly higher expression in FD+ groups as compared to all other infected groups (FD−, MD−, and MD+) (Figures 3(g) and S3(c)). Reduction of the CD28 expression is found in CD8T cells of diabetic males as compared to nondiabetic males, while an increase in its expression is observed in FD+ as compared to FD− (Figure 3(g)).

In conclusion, enhancement of Th1 and Th17 aggressive immune responses was observed in females with diabetes. It has to be noted here along with a higher percentage of Th17 cells, greater CD8T costimulation as marked CD28 expression [39], is found in the infected female diabetic groups than in the males (Figure 3(g)).

### 3.4. Interferon Gamma Expression within the T Cells Exhibits Higher Proinflammatory Activity in the Diabetic Female Group

We have assessed the cytokine expression by taking key proinflammatory cytokine Interferon-gamma (IFN*γ*). We assessed the expression status from the MFI within the permeabilized CD8T cells and CD4T cells. The IFN*γ*+ CD8T and CD4T cells were analyzed (Figure S4(a)–S4(c)) and IFN*γ* MFI was then calculated and represented in the bar diagram (Figures 3(h) and 3(i)). The female diabetic mice group (FD+) showed the highest expression of the proinflammatory cytokines within both CD8T cells (Figure 3(h)) and CD4T cells (Figure 3(i)). While a significant increase is observed in the FD+ CD8T cells as compared with FD− CD8T cells, no significant change in the MD+ was found.

In conclusion, along with a higher level of Th17 subsets of CD4T cells (Figure 3(f)), and enhanced CD8T cell activation (Figure 3(g)) there is also increased proinflammatory cytokines within CD4T and CD8T cells (Figures 3(h) and 3(i)) in diabetic female (FD+) group indicates an undergoing Th1/Th17 cell-mediated immune response.

### 3.5. Increase in Plasma Cells in Diabetic Males while the Increased Population of Memory B Cells as well as Immature B Cells Observed in the Female Diabetic Groups

**Figure 4:**
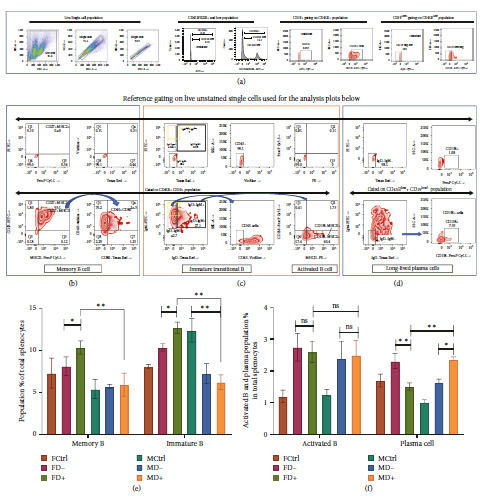
Determining population percentage of memory B cells, immature/transitional B cells, activated B cells, and plasma cells. (a) B220+ cells and B220^low^ populations are gated out from live single splenocyte suspension keeping the unstained sample as a reference for gating as usual. CD19+ cells are gated out from B220+ cells and CD19^low/neg^ cell populations are gated out from B220^low^ populations. (b) On the B220+CD19+ cell populations, CD27+MHCII+ populations are gated, on which CD40+CD80+ populations are determined to get B220/CD45R+CD19+CD27+MHCII+CD40+CD80+ memory B cells. (c) IgM^high^+IgD^low/neg^ population (yellow box) and IgM+IgD+ (thick black box) population are gated on B220+CD19+ populations. B220/CD45R+CD19+IgM^high^+IgD^low/neg^+CD43− immature/transitional B cells and B220/CD45R+CD19+IgM+IgD+MHCII+CD138− activated B cell population are determined. (d) From the B220^low^ CD19^low/neg^ populations IgM−IgD− cells are determined to obtain B220^low^ CD19^low/neg^ IgM−IgD−CD138+ plasma cells. (e) The bar diagram represents the memory B cells and immature/transitional B cells. (f) The bar diagram represents the activated B cells and plasma cells. The percentage exhibited in the *Y* axis of (e) and (f) is the percentage within the total 10,000 splenocytes analyzed. Hence, for comparison for absolute cell number, the conversion: 1% in the *Y* axis = 100 cells. D−, nondiabetic groups; D+, diabetic groups. Data in graphs are the representative images derived from at least four independent experiments ( ^*∗*^*p* < 0.05,  ^*∗∗*^*p* < 0.01, and  ^*∗∗∗*^*p* < 0.001). For each group *n* = 5, the error bar indicates the standard deviation.

Splenic live single-cell suspensions from all the groups were gated for different B-cell subsets [40, 41]. Cells were stained with B220 antibody to identify the B220^low^ and B220+ cell populations with respect to unstained cells (Figure 4(a)). The B220^low^ cells were then gated for CD19^low/neg^ and B220+ cells are gated for CD19+ cell populations using the unstained cells as reference (Figure 4(a)). B220/CD45R+CD19+ cells are gated for CD27+MHCII+ cells and CD40+CD80+ are gated on top of it to determine B220/CD45R+CD19+CD27+MHCII+CD40+CD80+ Memory B cells (Figure 4(b)). B220/CD45R+CD19+ cells are also gated for IgM^high^+IgD^low/neg^ population (yellow box) and IgM+IgD+ (thick black box) population. From there B220/CD45R+CD19+IgM^high^+IgD^low/neg^+CD43− immature/transitional B cells and B220/CD45R+CD19+IgM+IgD+MHCII+CD138− activated B cell population is identified (Figure 4(c)). B220^low^ CD19^low/neg^ IgM−IgD−CD138+ long-lived plasma Cells were also analyzed (Figure 4(d)).

An increase in memory B cells and immature/transitional B cells was observed in FD− as compared to FD+ (Figure 4(e)). A significant decrease in the immature/transitional B cells was observed in both the infected male groups with or without diabetes, while no significant change in the memory B cells was observed in them (Figure 4(e)). Although there were no significant changes in the activated B cells between the diabetic and nondiabetic group of both sexes, a significant drop in the plasma cells were observed in FD+ as compared to FD− (Figure 4(f)). A significant rise in the plasma cells was observed in diabetic males as compared to nondiabetic males and diabetic females (Figure 4(f)).

In conclusion, an increase in memory B and immature/transitional B cells with lower plasma cells in diabetic females as compared to nondiabetics provides a clue of the possibility of the suppressed humoral response. On the other side, a reduced population percentage of immature and memory B with a higher plasma cell population in diabetic males indicates a possible enhancement of humoral response.

### 3.6. Significantly Higher IgG1 Level in Plasma Cells of Diabetic Males while a Higher IgG2 Titer in Diabetic Females was Observed

**Figure 5:**
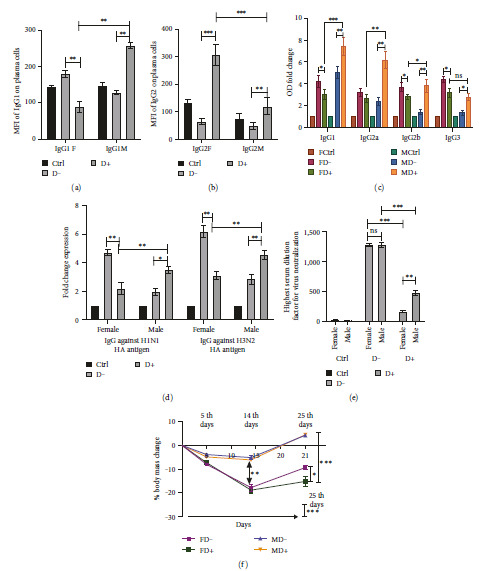
ELISA and FACS-based analysis of the immunoglobulin levels and subtypes indicates a drop in antibody response in the diabetic female while the same being elevated in the diabetic male. (a) The IgG1 level and (b) IgG2 level in the permeabilized B220^low^ CD19^low/neg^ IgM−IgD−CD138+ plasma cells were studied via FACS and represented in the bar diagram. (c) The antibody titer specific for anti-HA IgG1, IgG2a, IgG2b, and IgG3 were measured by ELISA with the plates coated with HA-specific for H1N1 for studying the serum IgG subtypes specific for the antigen. (d) Fold change expression for the antibody titers for anti-HA IgG specific for H1N1 and H3N2 hemagglutinin. (e) A hemagglutination inhibition assay was performed to evaluate the potential of the serum antibodies to neutralize the live influenza virus. The highest serum dilution factor that is still capable of neutralizing the virus is represented. (f) The alternation in body mass percentage as observed in 3 day-points that have been represented in the graph as 5-, 14-, and 25-days postinfection challenges. The statistical significance in the 14th day point represents for both MD+ versus MD− and FD+ versus FD−. The statistical significance bar for the 25th day indicates the significance for MD+ versus FD+. Data in graphs are derived from at least four independent experiments ( ^*∗*^*p* < 0.05,  ^*∗∗*^*p* < 0.01, and  ^*∗∗∗*^*p* < 0.001). For each group *n* = 5, the error bar indicates the standard deviation.

B220^low^CD19^low/neg^IgM−IgD−CD138+ plasma cells costained with the fluorescently stained IgG1 and IgG2 showed a marked reduction in the IgG1 with a significant increase of IgG2 in the FD+ as compared to FD− (Figures 5(a) and 5(b)). The same trend was observed in the number of IgG1+ and IgG2+ plasma cell population percentage (Figures S5(a) and S5(b)). A significant increase in IgG1 and IgG2 MFI is observed in the MD+ with respect to MD− (Figures 5(a) and 5(b)). The analysis of the whole spleen cell suspension also showed a similar trend (Figures S5(c) and S5(d)). Therefore, we conclude that in diabetes, IgG1 level is significantly higher in males as compared to females, while IgG2 levels are significantly higher in females compared to males (Figures 5(a) and 5(b)).

### 3.7. Influenza HA-Specific Serum IgG Subtyping Shows Both IgG1 and IgG2 Based Immune Responses with the Male Diabetic Group Exhibiting the Higher Titer

The serums isolated on the day of sacrifice are subjected to the ELISA for the detection of the anti-IgG antibodies specific to the coated influenza HA of H1N1. The anti-HA IgG antibody subtypes were studied. The significant reduction of IgG1, IgG2a, IgG2b, and IgG3 in the FD+ group as compared to FD− is observed. The opposite trend is true between MD+ and MD−, with a significant increase in MD+ observed (Figure 5(c)). In conclusion, the diabetic Male (MD+) group exhibits a significantly higher expression of IgG1, IgG2a, and IgG2b anti-HA antibodies with respect to diabetic females (FD+) (Figure 5(c)). No significant change is observed in IgG3 between these two groups (Figure 5(c)).

### 3.8. Enhanced Postchallenge Antibody Response Found in Diabetic Males than Diabetic Females

Serum collected on the day of sacrifice has been tested for anti-HA IgG response against H1N1 and H3N2 HA coated in the ELISA plates. The level of specific antibodies is significantly higher in the female in comparison to the male infected groups in nondiabetic conditions (Figure 5(d)). Females in the same group (FD−) showed stronger heterospecific antibody response against H3N2 HA, although all of them are provided with the H1N1 as vaccine antigen (Figure 5(d)). It is noteworthy that during diabetes the male produces a much stronger antibody response as derived from their IgG antibody titers in comparison to the FD+ group (Figure 5(d)). Thus, we conclude that although females have higher antibody titers in normal nondiabetic conditions, during diabetes the males showed significantly elevated humoral serum IgG response against vaccine antigen HA.

### 3.9. The Female Diabetic Group Shows Considerably Low Potency of Virus Neutralization than the Males

When we use the RDE-treated serum samples from each of the groups and investigated their virus neutralization capacity a clear picture emerges regarding the potency of these serums in combating the live virus. The diabetic male shows a strong virus neutralization almost like the normal male group (Figure 5(e)). The hemagglutination inhibition (HI) titer is the golden standard for providing a correlation with immune protection. HI titers ≥ 40 are accepted widely to correlate with 50% of immune protection against influenza [42]. Diabetic females performed worse than the males in the virus neutralization assay performed in this study. This correlates well with the drop in the HA-specific antibody in the ELISA (Figure 5(d)). It has to be noted that although normal nondiabetic females showed the highest antibody titer in the ELISA their neutralization potential is comparable with the nondiabetic males.

### 3.10. The Physical Parameter Signifies Enhanced Recovery in Diabetic Males after Viral Challenge although No Mortality was Observed in Both Sexes after Vaccination

The mice sets were followed up for all the groups and the body weight is measured as the indicator of health status. A significant reduction in body weight was observed in the diabetic groups both in males and females in comparison to the nondiabetic (Figure 5(f)). The male mice showed a faster recovery in their body weight after the infection challenge in both the diabetic and nondiabetic groups (MD+ and MD−) (Figure 5(f)). There was no mortality after the virus challenge in any of the vaccinated animal groups. They were sacrificed on the 31st-day postviral challenge. However, half of the control groups that were receiving the virus were all sacrificed within 12 days of the viral challenge and were excluded from the study.

## 4. Discussion

It is of utmost importance to design suitable vaccine strategies for specific diabetic groups. This is particularly important as there is an increased risk for influenza complications observed in patients with diabetes. They are ∼3x (95% CI: 2.04–4.71) more likely to be hospitalized [43], ∼4x (95% CI: 1.29–14.3) more likely to be admitted to the ICU [43], and ∼2x (95% CI: 1.5–3.6) more likely to die after influenza hospitalization [44, 45]. On average, diabetes deaths accounted for 19.6% of estimated influenza-associated all-cause mortality [46]. There are contradictory reports where some indicate no benefits from influenza vaccination in diabetes, while others found similar benefits for diabetes patients as that seen in healthy controls [18, 27–29]. However, the studies also reported biases in measuring the effectiveness of seasonal influenza vaccination in the diabetic patient group [18, 47]. The meta-analysis done on 1,444 related articles on this topic comprising 170,924 participants concluded about the lack of evidence to determine the magnitude of benefit that diabetic people are getting from the seasonal flu shots [18]. In this study, as there was no mortality in the vaccinated groups (MD−, MD+, FD−, and FD+) it can be safely concluded that both sexes are protected from the postvaccination influenza challenge, whether they are diabetic or not. The mechanism and efficiency level of protection varied between groups and their comparative summary has been provided in Table S2.

While comparing the FD− and FD+ groups, an increase in cDC1 (Figures 2(a), 2(b), and 2(d)), elevated expression of MHCI, CD80/86 positive costimulatory marker expression (Figures 2(e) and 2(f)), increase in CD8T cell population (Figure 3(c)), and its costimulation marker CD28 expression (Figure 3(f)) and proinflammatory activity of higher IFN*γ* level within CD8T cells (Figures 3(h) and 3(i)) all indicate an elevated CD8T immune pathway in diabetic females. Cell-mediated immunity was reported to be better correlated with influenza protection in persons with poor immune response [48, 49]. In humans, the females suffered most adversely from the influenza infection and have far higher symptoms [13, 14]. The significant decrease of cDC2 (Figures 2(c) and 2(d)), increase in immature B and memory B (Figure 4(e)), drop in plasma B cells (Figure 4(f)), drop in the influenza-specific antibody titers (Figures 5(c) and 5(d)), and virus neutralization capacity (Figure 5(e)) in the female diabetic group as compared to FD−, indicate a suppressed humoral response in diabetic condition with a switch in immune protection mode in diabetic females.

Previous reports in normal nondiabetic mouse models suggest the amount of H1N1 or H3N2 virus required to kill 50% of mice (LD50) was significantly lower in females as compared to males in C57BL/6 mice [15, 16]. It is 11-fold lower in the case of H1N1 infection and fourfold lower in the case of H3N2. The median dose of H1N1 constantly showed a greater reduction in body temperature, body mass, and survival of female mice as compared to males [15]. We find the same in FD− versus MD− comparison of body weight (Figure 5(f)). In the same study, Robinson et al. [15] reported higher mortality and morbidity in female C57BL/6 mice than the males after they are infected with influenza A virus A/PR/8/34 (PR8; H1N1). Therefore, even after having developed higher antibody titers in nondiabetic conditions, the females are doing far worse than the male after diabetes.

While comparing the MD− and MD+ groups, Diabetic males shift towards expressing abundant MHCII and DEC205 in cDC. DEC205 is a critical dendritic cell protein marker that takes the novel pathway of antigen uptake as they are recycled through MHCII-rich late endosomal compartments increasing antigen presentation to CD4+ helper T cells [50]. Apart from that MD+ group also showed the reduction of immature B cells and memory B cells (Figure 4(e)), with a significant increase in plasma cells (Figure 4(f)) producing higher influenza-specific antibody titers (Figures 5(c) and 5(d)) with enhanced neutralization capacity as compared to MD− group (Figure 5(e)). This indicates an enhanced humoral immunity in the diabetic male, which is stronger than the nondiabetic male and diabetic female.

The myeloid lineage conventional dendritic cells have two most common subsets, cDC1 and cDC2. It has been widely accepted that cDC1 is more efficient in the MHCI-mediated CD8T activation, while cDC2 is efficient in MHCII-mediated CD4T activation [51, 52]. In this study, the female diabetic group has a significantly higher cDC1 population percentage with increased MHCI expression, with an increase in CD8T cell (Figure 3(b)) and its activation (Figure 3(g)). The male diabetic group on the other hand has a significantly higher cDC2 population (Figure 2(c)), and better humoral response. The B cell development process is initiated from the bone marrow and after the negative selection, it migrates to a secondary lymphoid organ such as the spleen. The B cells in this naïve immature state express high IgM and very little to no IgD [40, 41]. The immature transitional B cells studied here (Figures 4(c) and 4(e)) significantly increased in diabetic females while lowered in infected male groups. However, after the antigen-dependent activation of the B cells to its activated state (Figures 4(c) and 4(e)) there were no significant differences during pre- and post-diabetic state within the same sex, although antibody-secreting long-lived plasma cells (Figures 4(d) and 4(f)) are significantly higher in male diabetic condition than the females.

The CD3+CD4+FoXP3+ regulatory T cells (Treg) facilitate Th2-based tolerogenic immune response [53, 54]. In case of a pathogenic challenge, it is quite normal to have higher CD3+CD4+RORgT+ Th17 Helper T cells which are more aggressive and drive Th1/Th17-based immune response [53, 54]. However, it has been seen that a balance between the two is most crucial to have a favorable outcome for the host. The Treg : Th17 ratio is an indicator of Th1 versus Th2 immune response where a drop in the ratio indicates a Th1 response [53, 54]. Significantly low Th17 cell population percentage in MD+ as compared to FD+, with no significant difference in Treg observed (Figures 3(e) and 3(f)) indicates a low Th1/Th17 response in MD+. Along with that, enhanced IgG1 level or titer in the MD+ as compared to FD+ also indicates an elevated Th2 response in them (Figures 5(a) and 5(c)). The whole IgG antibody titer specific to H1N1 and H3N2 HA antigen is also high in MD+ (Figure 5(d)). Elevated Th2 response is a better antibody production predictor [55, 56]. The humoral immune response elicited after vaccination is characterized by a Th2 response when the IgG antibody subclass distribution is dominated by IgG1. On the other hand, if it is dominated by the subclass IgG2, it indicates more of a Th1 response [57]. Higher IgG2 titer in the female diabetic group (Figure 5(b)) with a higher Th17 population, increased CD8T cell activation, and proinflammatory activities of significantly higher IFNg and TNFa levels as compared to MD+ (Figure 3(i)) all indicate Th1/Th17-type immune response. Controlled upregulation in Th1 response is necessary in the host in most cases for combating an infectious agent [56].

This activation of the humoral immune response pathways in male diabetic mice as observed in our study is exciting. In Type 1 diabetes, a higher antibody titer is reported in diabetic individuals before [17]. Alterations in blood glucose levels and metabolism have been reported to trigger the immune system in other diseases and conditions [58]. Sometimes they are related to hyperactivation of the immune system leading to cytokine storms [59] and also abundant antibody synthesis [60]. It has to be noted that in this study, both sexes exhibited the activation and immune response from both the immune arms. It is only that during diabetes the preferred immune protection mechanism of each sex got swapped than what they were in prediabetic condition.

In humans, half a dose of influenza vaccination in adult females elicits the same immune response as that derived from the full dose for males [28, 61]. Postvaccination, the females showed higher serum antibody titer specific to the influenza virus [28, 61] and provide enhanced protection against the heterospecific virus strains of influenza. We find the same in this study in mice model where nondiabetic females (FD−) showed a much stronger immune response with a much higher influenza HA− specific antibody response as compared to nondiabetic males (MD−). This FD− group also produced higher antibodies against the heterosubtypic strain of H3N2 (Figure 5(d)). This correlates with the previous findings from other groups [16, 62]. Lorenzo et al. [16] and L'Huillier et al. [62] reported that antibody responses and crossprotection against lethal influenza A viruses differ between the sexes in C57BL/6 mice. The potential molecular mechanism for these changes may stem from the innate difference in antiviral immunity shown by males and females. The differential processing of the antigens by the antigen-presenting cells of two sexes might also contribute toward the differential holistic downstream immune response observed.

The study is primarily focused on evaluating the vaccine efficacy in influenza infection during diabetes and not on the basic immunity difference between diabetic and nondiabetic groups. Hence, we choose not to include a diabetic group without immunization or viral challenge, that is diabetic control. The control that we have used in this study (MCtrl and FCtrl) is for having a sex-specific baseline comparison for every immune parameter studied. A limitation of this study is the lack of immune profile data at multiple time points after the infection and the vaccination. The immune response is a dynamic process that keeps on altering. So, to get a much broader and finer picture there is a need to study them at different time points in later studies and also to include other possible immune cells, chemokines, and cytokines in those future studies. To the limited scope of this study, we did not evaluate the potential influence of the female estrous cycle on local humoral immune response. However, since all the mice in female groups are of the same age range their influence on skewing the response will negate out within same-sex comparison. Another limitation to take into account is the reduced helper and cytotoxic T cell population in all the infected groups which might be due to T cell aging and senescence after long-term combat with the infection, as reported in other studies [38].

In conclusion, our study in the murine model successfully illustrates the mechanistic sex-specific difference of the seasonal vaccination-mediated immune protection against the influenza virus in diabetes. The results of this study could be interpreted to propose that vaccines should be designed or delivered to match the person's biological sex and comorbidity. This study also highlights the need of tailoring the existing FDA-approved vaccines to be necessary for better protection of the recipients [28]. Modification of the vaccine dose, adjuvant, mode of administration, types, and so forth can trigger the weak spot of each sex to confer much stronger protection for patients with severe complications. We strongly believe these mechanistic details of the alternative paths taken by each sex, their strength, and their limitations and weaknesses will be critical for shaping better sex-specific vaccine strategies in the future.

## Figures and Tables

**Table 1:** Illustrating the control and research groups (diabetes and nondiabetes) that were challenged by the influenza virus.

Group name	Gender	Infected by H1N1	Vaccine (VaxiGrip) + adjuvant N3	Number of individuals
Control (FCtrl)	Female	No	No	5
Nondiabetic (FD−)	Female	Yes	Yes	5
Diabetic (FD+)	Female	Yes	Yes	5
Control (MCtrl)	Male	No	No	5
Nondiabetic (MD−)	Male	Yes	Yes	5
Diabetic (MD+)	Male	Yes	Yes	5

*Note*: The groups in total were six; three males and three females groups. The study groups were vaccinated with VaxiGrip and the adjuvant N3 (1 *µ*g HA/N3) excluding the control. All vaccinated individuals in the research groups received the same dose. N3, cationic lipid adjuvant; H1N1 virus, influenza A/California/07/2009(H1N1)pdm.

## Data Availability

Most of the data generated or analyzed during this study are included in this published article (and its supplementary information files). All the datasets used and/or analyzed during the current study available from the corresponding author on reasonable request.

## Supplementary Materials

Supplementary Materials Table S1: Flow cytometry antibody combination used in the study along with the references. Figure S1: the absolute cell numbers for CD80+CD86+ cDC1 and CD80+CD86+ cDC2 are presented in the bar diagram. Figure S2: histogram plots of the cDC1 and cDC2 expressing MHCI and DEC205 are presented here with the MFI values in the table. Figure S3: the absolute number of CD3+CD4+FoXP3+ regulatory T cells (Treg), CD3+CD4+RoRgT Th17 cells, and the histogram plots with the MFI values of CD28 expression on CD3+CD8+ cytotoxic T cells have been plotted in the bar diagram. Figure S4: histogram plots for IFN*γ*+ CD8T and CD4T cells. Figure S5: the representative histogram plots from each group with IgG1+ plasma cells, IgG2+ plasma cells, IgG1+ whole splenocytes, and IgG2+ whole splenocytes are provided. Table S2: the comparative summary of the findings from this study.

## References

[1] Jacobsen H., Klein S. L. (2021). Sex differences in immunity to viral infections. *Frontiers in Immunology*.

[2] Takahashi T., Iwasaki A. (2021). Sex differences in immune responses. *Science*.

[3] Flanagan K. L., Fink A. L., Plebanski M., Klein S. L. (2017). Sex and gender differences in the outcomes of vaccination over the life course. *Annual Review of Cell and Developmental Biology*.

[4] Fischinger S., Boudreau C. M., Butler A. L., Streeck H., Alter G. (2019). Sex differences in vaccine-induced humoral immunity. *Seminars in Immunopathology*.

[5] Beery A. K., Zucker I. (2011). Sex bias in neuroscience and biomedical research. *Neuroscience & Biobehavioral Reviews*.

[6] Klein S. L. (2012). Immune cells have sex and so should journal articles. *Endocrinology*.

[7] World Health Organization (2010). *Sex, Gender and Influenza*.

[8] Serfung R. E., Sherman I. L., Houseworth W. J. (1967). Excess pneumonia-influenza mortality by age and sex in three major influenza A2 epidemics, United States, 1957–58, 1960 and 1963. *American Journal of Epidemiology*.

[9] Campbell A., Rodin R., Kropp R. (2010). Risk of severe outcomes among patients admitted to hospital with pandemic (H1N1) influenza. *Canadian Medical Association Journal*.

[10] Kumar A., Zarychanski R., Pinto R. (2009). Critically ill patients with 2009 influenza A (H1N1) infection in Canada. *JAMA*.

[11] Eshima N., Tokumaru O., Hara S. (2011). Sex- and age-related differences in morbidity rates of 2009 pandemic influenza A H1N1 virus of swine origin in Japan. *PLoS One*.

[12] Xi X., Xu Y., Jiang L., Li A., Duan J., Du B. (2010). Hospitalized adult patients with 2009 influenza A(H1N1) in Beijing, China: risk factors for hospital mortality. *BMC Infectious Diseases*.

[13] Morgan R., Klein S. L. (2019). The intersection of sex and gender in the treatment of influenza. *Current Opinion in Virology*.

[14] Giurgea L. T., Cervantes-Medina A., Walters K.-A. (2022). Sex differences in influenza: the challenge study experience. *The Journal of Infectious Diseases*.

[15] Robinson D. P., Lorenzo M. E., Jian W., Klein S. L. (2011). Elevated 17*β*-estradiol protects females from influenza A virus pathogenesis by suppressing inflammatory responses. *PLoS Pathogens*.

[16] Lorenzo M. E., Hodgson A., Robinson D. P., Kaplan J. B., Pekosz A., Klein S. L. (2011). Antibody responses and cross protection against lethal influenza A viruses differ between the sexes in C57BL/6 mice. *Vaccine*.

[17] Pozzilli P., Gale E. A. M., Visallil N. (1986). The immune response to influenza vaccination in diabetic patients. *Diabetologia*.

[18] Remschmidt C., Wichmann O., Harder T. (2015). Vaccines for the prevention of seasonal influenza in patients with diabetes: systematic review and meta-analysis. *BMC Medicine*.

[19] Klekotka R. B., Mizgała E., Król W. (2015). The etiology of lower respiratory tract infections in people with diabetes. *Advances in Respiratory Medicine*.

[20] Casqueiro J., Casqueiro J., Alves C. (2012). Infections in patients with diabetes mellitus: a review of pathogenesis. *Indian Journal of Endocrinology and Metabolism*.

[21] Ng K. W., Faulkner N., Cornish G. H. (2020). Preexisting and de novo humoral immunity to SARS-CoV-2 in humans. *Science*.

[22] Aziz F., Aberer F., Moser O. (2021). Impact of comorbidities on mortality in hospitalized influenza patients with diabetes—analysis of the Austrian Health Insurance. *Diabetes Research and Clinical Practice*.

[23] Valdés C., Unanue N., Hernández M. (2013). Is there a link between influenza and type I diabetes? Increased incidence of TID during the pandemic H1N1 influenza of 2009 in Chile. *Pediatric Endocrinology Reviews*.

[24] Nenna R., Papoff P., Moretti C. (2011). Detection of respiratory viruses in the 2009 winter season in Rome: 2009 influenza A (H1N1) complications in children and concomitant type 1 diabetes onset. *International Journal of Immunopathology and Pharmacology*.

[25] Gómez-Gómez A., Sánchez-Ramos E. L., Noyola D. E. (2021). Diabetes is a major cause of influenza-associated mortality in Mexico. *Revue d’Epidémiologie et de Santé Publique*.

[26] Shomali N., Mahmoudi J., Mahmoodpoor A. (2021). Harmful effects of high amounts of glucose on the immune system: an updated review. *Biotechnology and Applied Biochemistry*.

[27] Goeijenbier M., van Sloten T. T., Slobbe L. (2017). Benefits of flu vaccination for persons with diabetes mellitus: a review. *Vaccine*.

[28] Klein S. L., Pekosz A. (2014). Sex-based biology and the rational design of influenza vaccination strategies. *Journal of Infectious Diseases*.

[29] Martínez-Baz I., Navascués A., Portillo M. E. (2021). Effect of influenza vaccination in preventing laboratory-confirmed influenza hospitalization in patients with diabetes mellitus. *Clinical Infectious Diseases*.

[30] Hinkula J., Devito C., Zuber B. (2006). A novel DNA adjuvant, N3, enhances mucosal and systemic immune responses induced by HIV-1 DNA and peptide immunizations. *Vaccine*.

[31] Sengupta A., Azharuddin M., Cardona M. E. (2022). Intranasal coronavirus SARS-CoV-2 immunization with lipid adjuvants provides systemic and mucosal immune response against SARS-CoV-2 S1 spike and nucleocapsid protein. *Vaccines*.

[32] Sengupta A., Al-Otaibi N., Devito C., Lottersberger F., Hinkula J. (2022). Characterization of splenic and systemic immunity by differentially charged lipid adjuvants in enhancing post-intranasal immunization response against influenza.

[33] Li X.-Q., Chang D.-Y., Chen M., Zhao M.-H. (2019). Deficiency of C3a receptor attenuates the development of diabetic nephropathy. *BMJ Open Diabetes Research & Care*.

[34] Sengupta A., Mukherjee S., Ghosh S. (2020). Partial impairment of late-stage autophagic flux in murine splenocytes leads to SQSTM1/P62 mediated NRF2-KEAP1 antioxidant pathway activation and induced proteasome-mediated degradation in malaria. *Microbial Pathogenesis*.

[35] Palmer D. (1975). *Advanced Laboratory Techniques for Influenza Diagnosis, Repr. May 1979*.

[36] Pakalniškyte D., Schraml B. U. (2017). Chapter three - tissue-specific diversity and functions of conventional dendritic cells. *Advances in Immunology*.

[37] Naik S. H., Sathe P., Park H.-Y. (2007). Development of plasmacytoid and conventional dendritic cell subtypes from single precursor cells derived in vitro and in vivo. *Nature Immunology*.

[38] Tedeschi V., Paldino G., Kunkl M. (2022). CD8+ T cell senescence: lights and shadows in viral infections, autoimmune disorders and cancer. *International Journal of Molecular Sciences*.

[39] Linsley P. S., Ledbetter J. A. (1993). The role of the CD28 receptor during T cell responses to antigen. *Annual Review of Immunology*.

[40] LeBien T. W., Tedder T. F. (2008). B lymphocytes: how they develop and function. *Blood*.

[41] Hardy R. R., Hayakawa K. (2001). B cell development pathways. *Annual review of immunology*.

[42] Dunning A. J., DiazGranados C. A., Voloshen T., Hu B., Landolfi V. A., Talbot H. K. (2016). Correlates of protection against influenza in the elderly: results from an influenza vaccine efficacy trial. *Clinical and Vaccine Immunology*.

[43] Allard R., Leclerc P., Tremblay C., Tannenbaum T.-N. (2010). Diabetes and the severity of pandemic influenza A (H1N1) infection. *Diabetes Care*.

[44] Ruiz P. L. D., Tapia G., Bakken I. J. (2018). Pandemic influenza and subsequent risk of type 1 diabetes: a nationwide cohort study. *Diabetologia*.

[45] Wilking H., Buda S., von der Lippe E. (2010). Mortality of 2009 pandemic influenza A(H1N1) in Germany. *Eurosurveillance*.

[46] Gómez-Gómez A., Sánchez-Ramos E. L., Noyola D. E. (2021). Diabetes is a major cause of influenza-associated mortality in Mexico. *Revue d’Épidémiologie et de Santé Publique*.

[47] Casanova L., Gobin N., Villani P., Verger P. (2016). Bias in the measure of the effectiveness of seasonal influenza vaccination among diabetics. *Primary Care Diabetes*.

[48] McElhaney J. E., Ewen C., Zhou X. (2009). Granzyme B: correlates with protection and enhanced CTL response to influenza vaccination in older adults. *Vaccine*.

[49] Ewen K. P., Kane R. C., Bleackley B. (2006). T cell responses are better correlates of vaccine protection in the elderly. *The Journal of Immunology*.

[50] Mahnke K., Guo M., Lee S. (2000). The dendritic cell receptor for endocytosis, DEC-205, can recycle and enhance antigen presentation via major histocompatibility complex class II-positive lysosomal compartments. *The Journal of Cell Biology*.

[51] Merad M., Sathe P., Helft J., Miller J., Mortha A. (2013). The dendritic cell lineage: ontogeny and function of dendritic cells and their subsets in the steady state and the inflamed setting. *Annual Review of Immunology*.

[52] Durai V., Murphy K. M. (2016). Functions of murine dendritic cells. *Immunity*.

[53] Ren J., Li B. (2017). The functional stability of FOXP3 and ROR*γ*t in Treg and Th17 and their therapeutic applications. *Advances in Protein Chemistry and Structural Biology*.

[54] Mukherjee S., Ghosh S., Bhattacharyya A. (2021). Regulation of T-Reg/Th-17 balance: one step closer towards immunotherapy against malaria infection. *Plasmodium Species and Drug Resistance*.

[55] Infante-Duarte C., Kamradt T. (1999). Th1/Th2 balance in infection. *Springer Seminars in Immunopathology*.

[56] Spellberg B., Edwards J. E. (2001). Type 1/type 2 immunity in infectious diseases. *Clinical Infectious Diseases*.

[57] Hauge S., Madhun A. S., Cox R. J., Brokstad K. A., Haaheim L. R. (2007). A comparison of the humoral and cellular immune responses at different immunological sites after split influenza virus vaccination of mice. *Scandinavian Journal of Immunology*.

[58] Zhang P., Yang C.-L., Du T. (2021). Diabetes mellitus exacerbates experimental autoimmune myasthenia gravis via modulating both adaptive and innate immunity. *Journal of Neuroinflammation*.

[59] Wang Q., Fang P., He R. (2020). *O*-GlcNAc transferase promotes influenza a virus-induced cytokine storm by targeting interferon regulatory factor-5. *Science Advances*.

[60] Gamble D. R., Kinsley M. L., FitzGerald M. G., Bolton R., Taylor K. W. (1969). Viral antibodies in diabetes mellitus. *British Medical Journal*.

[61] Engler R. J. M., Nelson M. R., Klote M. M. (2008). Half- vs full-dose trivalent inactivated influenza vaccine (2004–2005): age, dose, and sex effects on immune responses. *Archives of Internal Medicine*.

[62] L’Huillier A. G., Ferreira V. H., Hirzel C. (2020). T-cell responses following natural influenza infection or vaccination in solid organ transplant recipients. *Scientific Reports*.

[63] Sengupta A., Al-Otaibi N., Hinkula J. (2022). Sex-specific switching of responsive immune pathways in vaccinated diabetic murine model exposed to influenza infection.

