# Supplementary material for: Sex-Specific Immune Responses to Seasonal Influenza Vaccination in Diabetic Individuals: Implications for Vaccine Efficacy
Source: J Immunol Res. 2023 Oct 17;2023:3111351. doi: 10.1155/2023/3111351 (PMC10597737; doi:10.1155/2023/3111351)
Supplement: Supplementary Materials — Table S1: Flow cytometry antibody combination used in the study along with the references. Figure S1: the absolute cell numbers for CD80+CD86+ cDC1 and CD80+CD86+ cDC2 are presented in the bar diagram. Figure S2: histogram plots of the cDC1 and cDC2 expressing MHCI and DEC205 are presented here with the MFI values in the table. Figure S3: the absolute number of CD3+CD4+FoXP3+ regulatory T cells (Treg), CD3+CD4+RoRgT Th17 cells, and the histogram plots with the MFI values of CD28 expression on CD3+CD8+ cytotoxic T cells have been plotted in the bar diagram. Figure S4: histogram plots for IFNγ+ CD8T and CD4T cells. Figure S5: the representative histogram plots from each group with IgG1+ plasma cells, IgG2+ plasma cells, IgG1+ whole splenocytes, and IgG2+ whole splenocytes are provided. Table S2: the comparative summary of the findings from this study. [file 3111351.f1.docx]

**The supplementary description:**

**S1)** the details of the methods we used in the study including two tables: Supp. Table 1: the antibody list used for the study.

**S2)** Supporting Figures of the data.

**SUPPLEMENTARY**

**S1: Supplementary Methods:**

**Flow cytometry:**

Euthanized mice were sacrificed on the 31^st^ -day post-infection and spleens were harvested out. Mechanical tapping of the spleen and passing the cells through cell strainers were done to make the single-cell suspension of the splenocytes. The cells were washed with phosphate buffer saline (PBS) and centrifuged at 1000 rpm for 10 min at 4°C and the supernatant was discarded. The cell pellet was then resuspended in 2ml RBC Lysis Buffer for getting rid of the RBC in the solution. They were incubated for 5 minutes at room temperature. After that, the cells were centrifuged for 10 min at 4°C at 1000 rpm, and the pellet was resuspended in PBS for washing. The washing was repeated for three times. The cells were finally resuspended in cell staining buffer (CSB) containing 2% FBS in PBS. The cells were first blocked by incubating them with anti-CD16/CD32 followed by two washing steps with PBS.

The cells were then aliquoted and incubated with the fluorochrome-conjugated antibodies (Milteny Biotech) according to the combination necessary for experiment requirement by following the manufacturer’s protocol for antibody dilution, duration of incubation, washing etc. For intracellular staining the permeabilization of the cells were done by the 0.1-0.3% Triton™ X-100 followed by fixation by using 4% paraformaldehyde followed by washing with PBS. Side scatter and Forward scatter gate reference is used to determine the viable or live cell population. Isotype-matched antibodies (Milteny biotech) were used as staining controls. Unstained cells are used to understand signal from each antibody and to adjust the compensation matrix. The data compensation for the fluorescence spill over to other filters was taken care of by the software gating. Fluorescence signals from the labelled cells were acquired using a BD AriaIII Machine and analysed by Flow Jo software.

**Supplementary Table 1:**

| **Flow Cytometry Antibody combination used** | **Phenotypes/Activation marker** | **Antibodies used*** | **References** (below this table) |
| --- | --- | --- | --- |
| **Dendritic Cells** | | B220- APC-Vio770  MHCII- FITC  CD11c-APC  CD11b- PE-Vio615  CD8a-PerCP-Vio700  CD80- VioBlue  CD86- PE  MHCI- Vioblue  DEC205-PE | 1,2 |
| B220-MHCII+ CD11c+CD11b- CD8a+ | Conventional Dendritic cells 1 (cDC1) |  |  |
| B220-MHCII+ CD11c+CD11b+ CD8a- | Conventional Dendritic cells 2 (cDC2) |  |  |
| cDC1/cDC2 + CD80+ CD86 | costimulatory expression |  |  |
| cDC1/cDC2 +MHCI | MHC / costimulation marker |  |  |
| cDC1/cDC2 +DEC205 | Activation/ costimulation marker |  |  |
| **T cells:** | | CD3- PerCP-Vio700  CD8- APC-Vio770  CD4- PE-Vio615  FOXP3-PE  RORgT- APC  CD28-PE  INFg-PE | 3, 4 |
| CD3+CD8+ | Cytotoxic T cells |  |  |
| CD3+CD8+CD28 | costimulatory expression |  |  |
| CD3+CD4+ | Helper T cells |  |  |
| CD3+CD4+ FOXP3+ | Treg subset of CD4T cells |  |  |
| CD3+CD4+ RORγT+ | Th17 subset of CD4T cells |  |  |
| **B cells:** | | B220-APC  CD19- APC-Vio770  MHCII-PerCP-Vio700  MHCII- PE  IgM- FITC  IgD- PE-Vio615  CD138- PerCP-Vio700  CD40- Vioblue  CD80- PE-Vio615  CD43- Vioblue  CD27-FITC | 5, 6 |
| *B220^low^ CD19 ^low/neg^ IgM-IgD-CD138+* | Long-lived Plasma cells |  |  |
| *B220/CD45R+CD19+ IgM+IgD+MHCII+CD138-* | Activated B cell |  |  |
| *B220/CD45R+CD19+ IgM^high^+IgD^low/neg^+CD43-* | Immature/Transitional B |  |  |
| CD45R/B220+CD19+ CD27+MHC2+CD80+CD40+ | Memory B cells |  |  |

**Supp. Table 1: Flow Cytometry Antibody combination used in the study along with the References**

*Flurochrome tags mentioned are the laser fluroschorme used in the BD ARIAIII machine. For example, Alexa Fluor 488 tagged antibody is mentioned as FITC as FITC-filter in the FACS machine have been used and mentioned throughout the manuscript.

**REFERENCES for Supp Table 1:**

1. <https://www.miltenyibiotec.com/SE-en/resources/macs-handbook/mouse-cells-and-organs/mouse-cell-types/dendritic-cells-mouse.html>
2. <https://www.biolegend.com/en-us/dendritic-cells>
3. Golubovskaya V, Wu L. Different Subsets of T Cells, Memory, Effector Functions, and CAR-T Immunotherapy. Cancers (Basel). 2016;8(3):36. Published 2016 Mar 15. doi:10.3390/cancers8030036
4. <https://www.abcam.com/primary-antibodies/effector-t-cell-markers>
5. <https://resources.rndsystems.com/images/site/rnd-systems-bcells-br.pdf>
6. <https://www.bdbiosciences.com/content/dam/bdb/marketing-documents/Bcell_Brochure.pdf>

**2.5 Serology screening**

Blood samples (0.5 ml) were collected from all the animal groups of Table 1 on the day of sacrifice. We have also collected serum after the first and second vaccine dose to check the serum level of the antibody produce before the virus challenge. Serum (0.15 ml) was separated following the standard procedure as described before [31]. For serum screening, 96-well microplates (Nunclon, Copenhagen, Den-mark) were coated with 0.5 µg/mL recombinant H1N1 or H3N2 Hemagglutinin (HA) antigen (as according to experiment requirement) in sterile PBS and incubated 4°C overnight. Serum samples were diluted in PBS 2.5 % fat-free milk buffer with 0.05 % Tween 20. Serial dilutions from 1:100 to 1:100,000 were prepared and 100 µl from each dilution were transferred in duplicate wells into antigen coated plates, followed by incubation at 37oC for 90 min. Thereafter, plates were rinsed with PBS+0.05% Tween 20 (PBS-T). HRP-labelled conjugate goat-anti-mouse IgG (BioRad, Richmond, CA) was added to each well (100 µl/well) and incubated at 37oC for 90 min. For the IgG subtyping, anti-mouse IgG1, IgG2a, IgG2b and IgG3 antibodies are used followed by anti-goat IgG-HRP antibody. Then, plates were washed again with PBS-T, followed by addition of 0.2 g o-phenylenediamine (OPD) (from Sigma-Aldrich, S:t Louis, MA) to 0.03% H2O2 in 1ml. Then, plates were incubated in dark for 30 min at room temperature. The reaction termination was done by adding 100 µl of 2.5 % H2SO4 to each well. The absorbance was measured at OD in ELISA plate reader (Molecular Devices, Spectramax ID3). Cut off value for positive reactivity was calculated from the mean OD490 plus 3SD for negative control samples.

**2.5 Hemagglutination inhibition assay.**

The hemagglutination inhibition assay (HAI) was used to evaluate the presence of neutralizing anti-HA antibodies against viral influenza A in serum from individual mice as described previously [34]. An HI titer≥ 40 was defined as a protective amount of serum antibodies. In short, serum from individual mice was treated with receptor-destroying enzyme (RDE) at 37°C overnight. This is to remove non-specific serum HAI inhibitors. RDE was inactivated by incubation for 30 min at 56°C followed by the addition of 350µl NaCl 0.9%. The HAI assay was initiated by adding 25µl PBS to each well of a microtitre plate, followed by the addition of 50µl of receptor-destroying enzyme (RDE) treated serum. Serum was diluted in 8-fold serial dilutions starting from 1/10 up to 1/1280 dilutions. 25µl of influenza A/H1N1/CA09pdm containing 4 haemagglutinating units (HU) was added in to each well. The plate was shaken, covered, and incubated for 15 min at 20-25°C. Subsequently, 50µl chicken erythrocytes were added, and mixed, followed by incubation at 4°C for one hour. Thereafter, the plate was evaluated for hemagglutination and the degree of hemagglutination inhibition (HAI). The highest dilution fold of serum activity and neutralizations are noted to understand serum potency of virus neutralization.

**S2: SUPPLEMENTARY FIGURES**

**Supplementary FIGURE 1:**


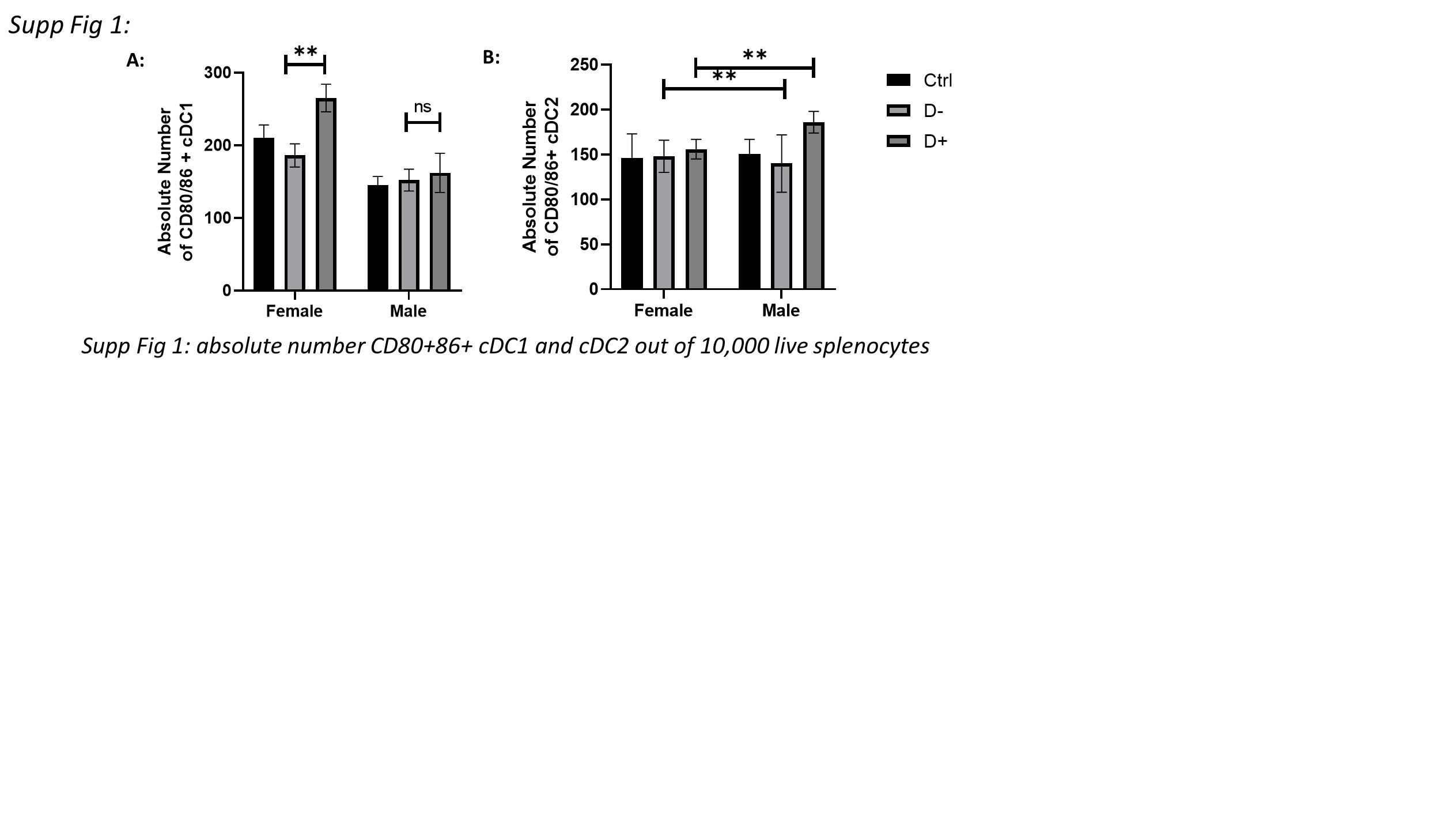


**Supplementary Figure 1:** A) The absolute cell number for CD80+CD86+ cDC1 are presented in the bar diagram. B) The absolute cell number for CD80+CD86+ cDC2 are presented in the bar diagram. *Data in graphs are the representative images derived from at least four independent experiments (*p < 0.05, **p < 0.01 and ***p < 0.001).*


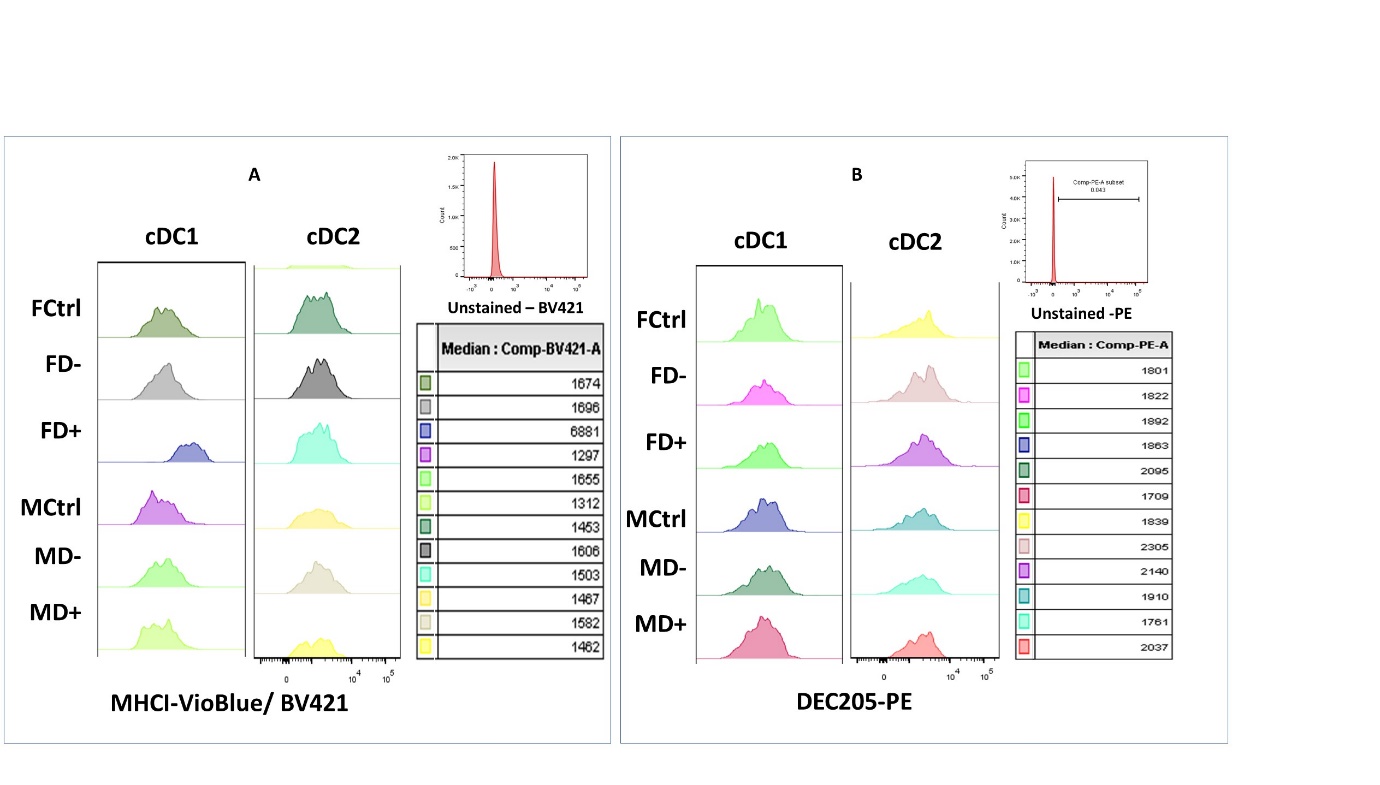


**Supplementary FIGURE 2:**

**Supplementary Figure 2:** Histogram plots of the cDC1 and cDC2 expressing A)MHCI and B)DEC205 are presented here with the MFI values in the table. These are the representative plots from individual groups that have been studied.


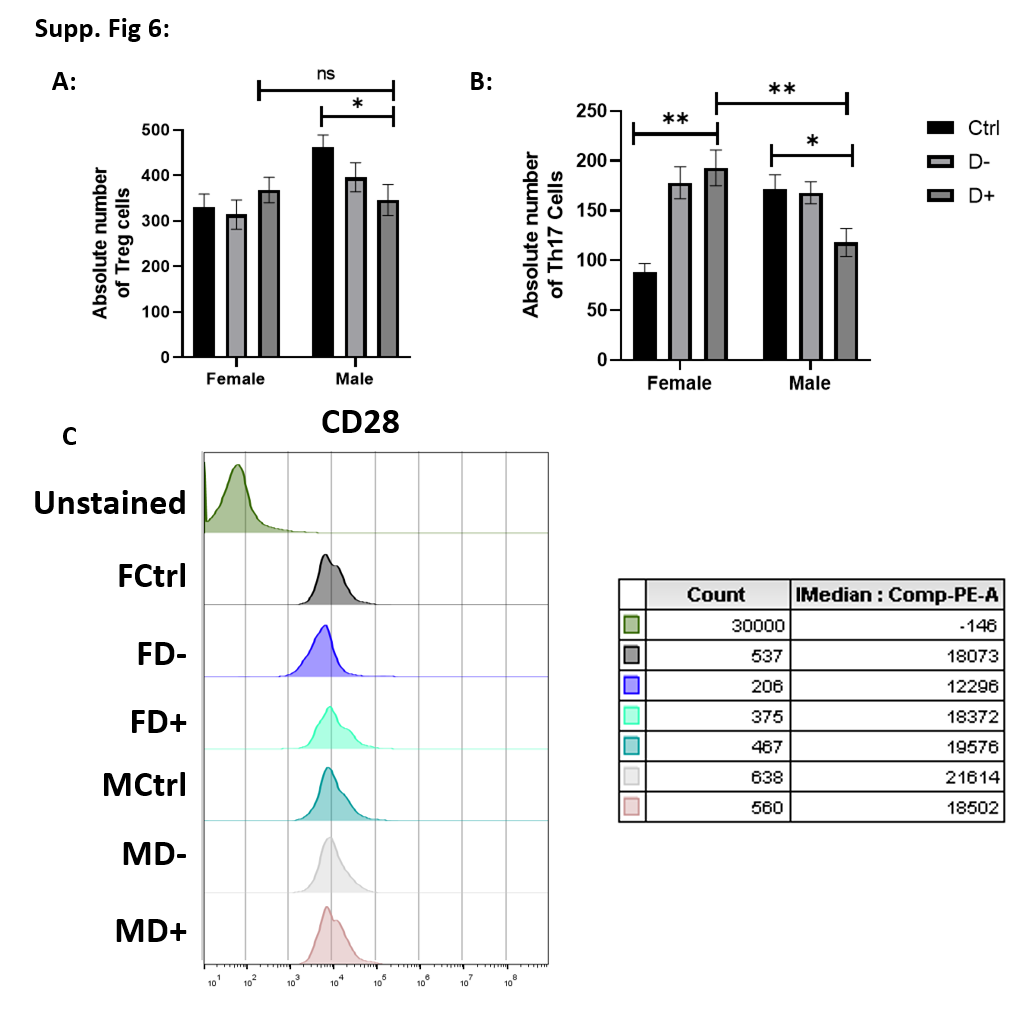


**Supplementary FIGURE 3:**

**Supplementary Figure 3:** A) Absolute number of CD3+CD4+FoXP3+ regulatory T cells (Treg) have been plotted in the bar diagram. B) Absolute number of CD3+CD4+RoRgT Th17 cells have been plotted in the bar diagram. C) The histogram plots with the MFI values of CD28 expression on CD3+CD8+ cytotoxic T cells are presented. These are the representative plots from individual groups that have been studied. *Data in graphs are the representative images derived from at least four independent experiments (*p < 0.05, **p < 0.01 and ***p < 0.001).*

**Supplementary FIGURE 4:**


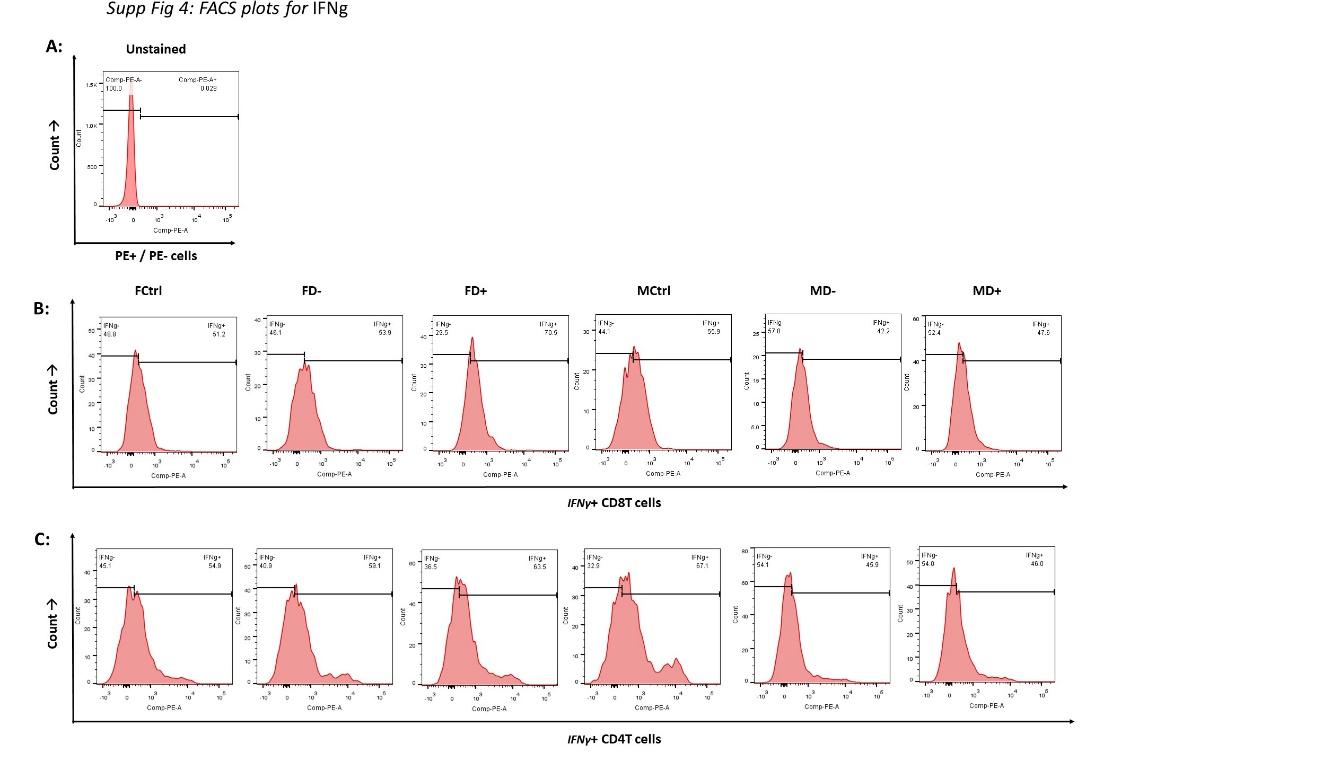


**Supplementary Figure 4:** Histogram plots for IFNγ+ CD8T and CD4T cells. A) the unstained live cells are provided. Representative histogram plots from each groups are provided in B) IFNγ+ CD8T cells and C) IFNγ+ CD4T cells.

**Supplementary FIGURE 5:**


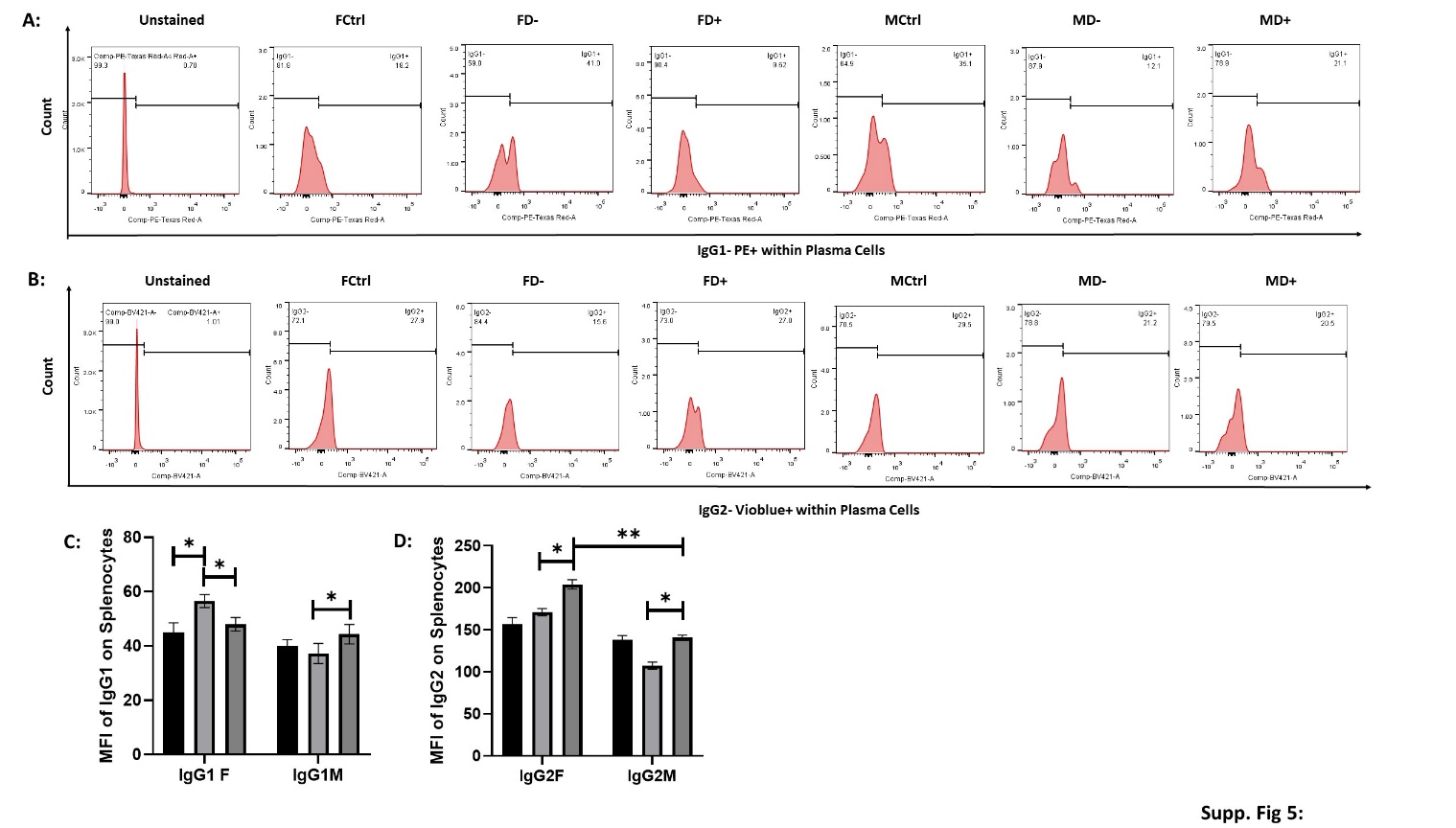


**Supplementary Figure 5:** The representative histogram plots from each group with A) IgG1+ plasma cells and B) IgG2+ plasma cells. The MFI for C) IgG1+ whole splenocytes and D) IgG2+ whole splenocytes are provided. *Data in graphs are the representative images derived from at least four independent experiments (*p < 0.05, **p < 0.01 and ****
